# Neonatal inguinal hernias containing the uterus: a case report on changes in uterine position

**DOI:** 10.1093/jscr/rjad503

**Published:** 2023-09-05

**Authors:** Yuki Muta, Akio Odaka, Seiichiro Inoue, Yuta Takeuchi, Yoshifumi Beck

**Affiliations:** Department of Hepato-Biliary-Pancreatic and Pediatric Surgery, Saitama Medical Center, Saitama Medical University, Saitama 3508550, Japan; Department of Hepato-Biliary-Pancreatic and Pediatric Surgery, Saitama Medical Center, Saitama Medical University, Saitama 3508550, Japan; Department of Hepato-Biliary-Pancreatic and Pediatric Surgery, Saitama Medical Center, Saitama Medical University, Saitama 3508550, Japan; Department of Hepato-Biliary-Pancreatic and Pediatric Surgery, Saitama Medical Center, Saitama Medical University, Saitama 3508550, Japan; Department of Hepato-Biliary-Pancreatic and Pediatric Surgery, Saitama Medical Center, Saitama Medical University, Saitama 3508550, Japan

**Keywords:** female, sliding inguinal hernia, uterus

## Abstract

A female newborn weighing 542 g and delivered at 27 weeks gestation presented with bilateral inguinal hernias while in the neonatal intensive care unit. Ultrasonography confirmed herniation of the uterus into the right inguinal hernia without signs of incarceration. Due to the absence of complications, she was discharged and scheduled for follow-up at the outpatient clinic. At 11 months of age, a subsequent ultrasonography showed only omental herniation, with no evidence of uterine prolapse. When she reached 1 year of age, a laparoscopic percutaneous extraperitoneal closure procedure was performed. During the surgery, it was observed that the uterus and fallopian tubes were located near the hernia orifice, but no clear prolapse was detected. The procedure concluded safely with successful high ligation. It has noted that in cases of uterine prolapse hernias, the uterus tends to recede as the child grows, which supports the decision to delay surgery for improved safety.

## Introduction

The occurrence of inguinal hernias in childhood ranges from 0.8% to 4% [1]. Approximately 15%–20% of inguinal hernias include the fallopian tubes and ovaries as sliding components [2, 3]. However, the presence of a uterus-containing inguinal hernia, where the uterus and its adnexa are involved, is exceptionally rare in female infants [4].

### Case report

A female newborn weighing 542 g was delivered via emergency cesarean section at 27 weeks of gestation due to concerns about the fetus’s well-being. Due to her extremely low birth weight, she was admitted to the neonatal intensive care unit (NICU) and required respiratory and circulatory support immediately after birth. Chromosomal analysis using G-banding indicated a normal karyotype of 46, XX without any observable abnormalities. As the infant’s condition stabilized and she gained weight, she was discharged from the NICU on postnatal day 183, weighing 2928 g.

**Figure 1:**
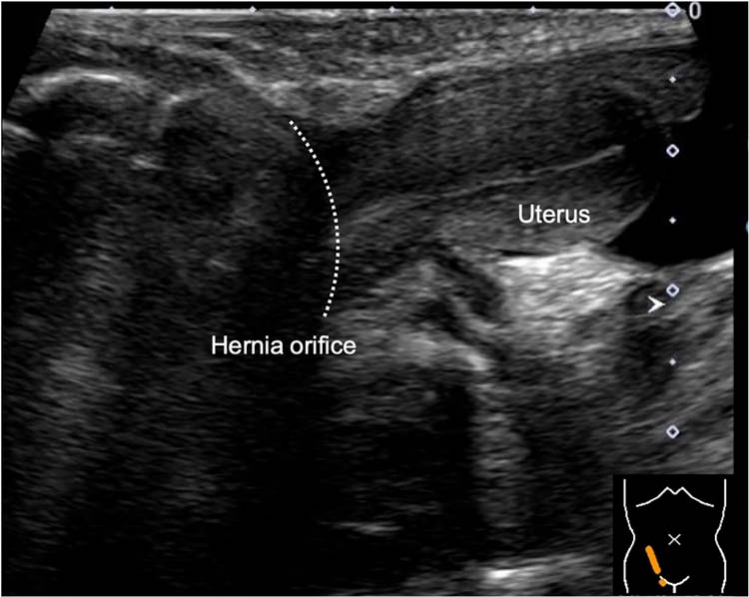
Sagittal view of the inguinal ultrasound at 3 months of age, revealing the presence of uterus protruding into the hernial sac.

**Figure 2:**
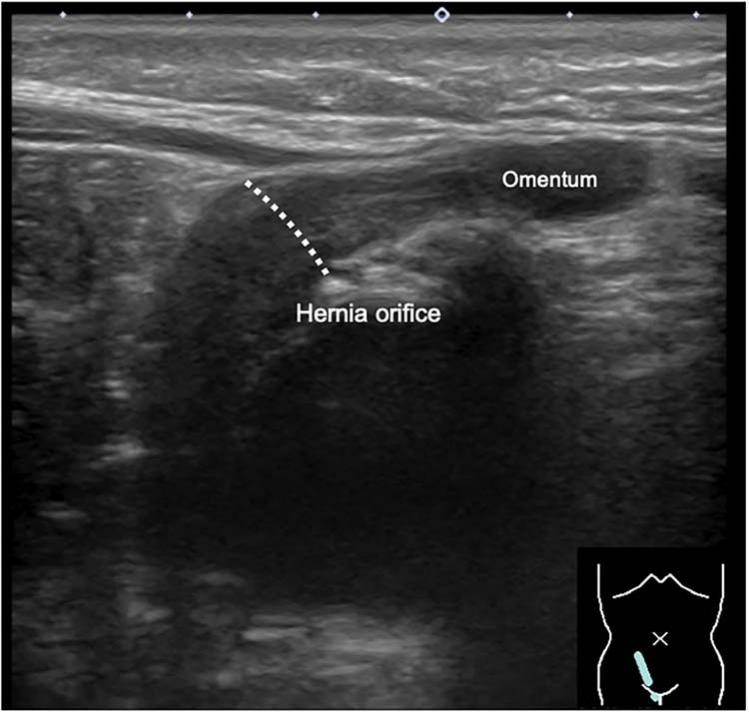
Inguinal ultrasound image captured at 11 months of age, showing herniation of the omentum but no apparent protrusion of the uterus.

During the newborn’s stay in the NICU, both inguinal regions showed swelling. An ultrasound examination conducted when the infant was 3 months old revealed bilateral inguinal hernias, with the uterus herniating on the right side (Fig. 1). There were no signs of incarcerated hernia, and the urgency of the situation was considered low. As a result, the patient was discharged from the NICU and received outpatient care. At 11 months of age, ultrasound examination was performed, which revealed the presence of omental herniation but no evidence of uterine prolapse (Fig. 2).

**Figure 3:**
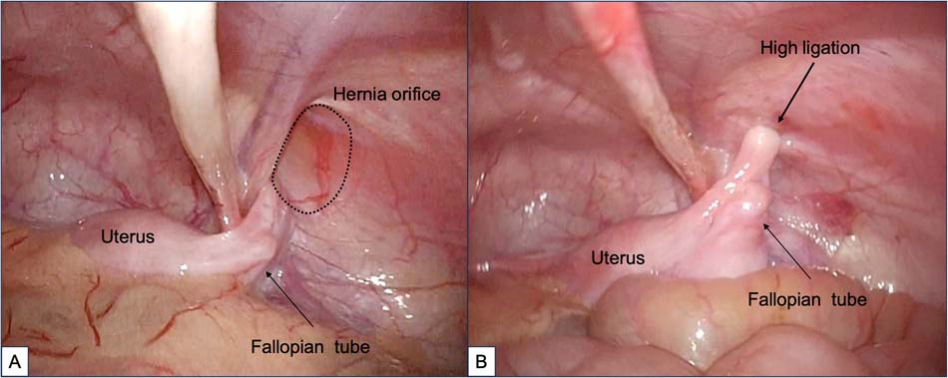
Findings during laparoscopic inguinal hernia repair (Laparoscopic Percutaneous Extraperitoneal Closure: LPEC) conducted at the age of 1 year. (A). Preoperative observations: The patent right inguinal hernia orifice was confirmed. The uterus and fallopian tubes were located close to the hernia orifice, but there was no evident protrusion, and the uterus was not in a position to prolapse. (B). Postoperative observations: High ligation was safely performed, with special attention given to the fallopian tubes.

When the patient turned 1 year old, a laparoscopic percutaneous extraperitoneal closure (LPEC) procedure was carried out to address the bilateral inguinal hernias. Although the uterus was slightly deviated toward the right side of the pelvis, its position appeared to make it difficult for the uterus to herniate through the hernia orifice. The right fallopian tube was located near the hernia orifice, and a high ligation was carefully performed to avoid entanglement of the fallopian tube (Fig. 3). The procedure was concluded successfully without any significant complications. Subsequent follow-up examinations conducted after one year showed no recurrence of the inguinal hernia.

## Discussion

We encountered a case involving a uterus-containing inguinal hernia in an infant and observed its natural progression. As the child matured, the prolapse of the uterus diminished. This resolution of the hernia was believed to be due to the improved positioning of the uterus toward the previously herniated side, which had exhibited a tilt, as a result of the child’s aging and growth [5].

Sliding hernias involve the displacement of various organs, including the ipsilateral ovary, fallopian tube, and even the uterus. The inguinal canal has the capacity to accommodate different intra-abdominal organs, such as the omentum, small intestine, appendix, Meckel’s diverticulum, ovary, fallopian tubes, and bladder. In female infants, sliding inguinal hernias can occur, affecting the ipsilateral ovary and fallopian tube, and there have been reported cases involving both ovaries. It is important to note that the uterus can also become trapped within these hernias [3, 4]. The incidence of sliding inguinal hernias is highest among preterm and female infants under 1 year of age, with the frequency decreasing as they grow older. According to reports, the occurrence of inguinal hernias containing the ovary and fallopian tubes in infants <2 years of age ranges from 15% to 30% [1].

The presence of the uterus within the hernial sac is typically observed in individuals who are hermaphrodites or intersexual variants or have a male phenotype (46, XY). Male pseudo-hermaphroditism, which involves the presence of Müllerian duct derivatives such as the uterus, cervix, fallopian tubes, and upper one-third of the vagina, can occur in phenotypic males [6–8]. In children with a female phenotype, the occurrence of a sliding hernia involving the uterus is an extremely rare condition, and its frequency is unknown [4]. Symptoms may include inguinal swelling, reducible or incarcerated inguinal hernias, and vaginal bleeding. It is important to consider the presence of the uterus as a sliding component of the hernia when vaginal bleeding is observed in a child with an inguinal hernia [2, 3, 6, 9]. Although the embryological cause of this condition is not fully understood, elongated uterine suspensory ligaments have been proposed as a contributing factor. While adult women with herniated uterus and inguinal involvement often exhibit various genital abnormalities, no such anomalies were found in this particular case. Preoperative ultrasound scans can assist in diagnosis and determining the appropriate surgical treatment, although they were not performed in this limited series [4].

Inguinal hernia repair is a commonly performed surgical procedure in pediatric patients, and the standard approach involves high ligation of the hernial sac [3]. However, there have been reported cases suggesting a potential link between pediatric inguinal hernia surgery (with high ligation) during childhood and tubal occlusion [10]. Although the presence of ovaries and fallopian tubes on both sides implies that unilateral tubal occlusion may not lead to infertility symptoms, it is believed that there may be a greater number of cases with tubal occlusion following inguinal hernia surgery than currently recognized [10]. As the child grows, the deviation of the uterus toward the hernia side resolves, causing the uterus and its adnexa to move further away from the hernial orifice [5]. Therefore, by adopting a strategy of carefully monitoring the progress through ultrasound while being cautious of incarceration and opting for delayed surgery, it is possible to achieve resolution of uterine and adnexal prolapse, thereby reducing the risk of complications such as tubal occlusion associated with inguinal hernia surgery. Furthermore, laparoscopic inguinal hernia repair (including LPEC) allows for direct visualization of the ovaries, fallopian tubes, and uterus, enabling high ligation while observing these structures, thus further minimizing the possibility of tubal occlusion [11].

Uterus-containing inguinal hernia is an extremely rare, and ultrasound examination is useful for its diagnosis. When there is no incarceration, the presence of uterine prolapse alone does not require immediate surgical intervention. We consider that waiting for growth would resolve the uterine prolapse, leading to a higher likelihood of performing a safer surgical procedure.

## Conflict of interest statement

The authors declare no conflict of interest.

## Funding

None declared.
